# Plant-Mediated Silencing of the Whitefly *Bemisia tabaci* Cyclophilin B and Heat Shock Protein 70 Impairs Insect Development and Virus Transmission

**DOI:** 10.3389/fphys.2019.00557

**Published:** 2019-05-08

**Authors:** Surapathrudu Kanakala, Svetlana Kontsedalov, Galina Lebedev, Murad Ghanim

**Affiliations:** Department of Entomology, Agricultural Research Organization, The Volcani Center, Rishon LeZion, Israel

**Keywords:** *Bemisia tabaci*, silencing, Cyclophilin B, Hsp70, *Tobacco rattle virus*

## Abstract

The whitefly *B. tabaci* is a global pest and transmits extremely important plant viruses especially begomoviruses, that cause substantial crop losses. *B. tabaci* is one of the top invasive species worldwide and have developed resistance to all major pesticide classes. One of the promising alternative ways for controlling this pest is studying its genetic makeup for identifying specific target proteins which are critical for its development and ability to transmit viruses. *Tomato yellow leaf curl virus* (TYLCV) is the most economically important and well-studied begomovirus transmitted by *B. tabaci*, in a persistent-circulative manner. Recently, we reported that *B. tabaci* Cyclophilin B (*CypB*) and heat shock protein 70 proteins (*hsp70*) interact and co-localize with TYLCV in the whitefly midgut, on the virus transmission pathway, and that both proteins have a significant role in virus transmission. Here, we extended the previous work and used the *Tobacco rattle virus* (TRV) plant-mediated RNA silencing system for knocking down both genes and testing the effect of their silencing on whitefly viability and virus transmission. Portions of these two genes were cloned into TRV constructs and tomato plants were infected and used for whitefly feeding and transmission experiments. Following whitefly feeding on TRV-plants, the expression levels of *cypB* and *hsp70* in adult *B. tabaci* significantly decreased over 72 h feeding period. The knockdown in the expression of both genes was further shown in the first generation of silenced whiteflies, where phenotypic abnormalities in the adult, wing, nymph and bacteriosomes development and structure were observed. Additionally, high mortality rates that reached more than 80% among nymphs and adults were obtained. Finally, silenced whitefly adults with both genes showed decreased ability to transmit TYLCV under lab conditions. Our results suggest that plant-mediated silencing of both *cypB* and *hsp70* have profound effects on whitefly development and its ability to transmit TYLCV.

## Introduction

Insect vectors of plant pathogens are distributed worldwide and are the driving force for disseminating more than 70% of the plant viruses worldwide (Hogenhout et al., 2008). The whitefly *Bemisia tabaci* is a supervector and transmits more than 100 plant viruses, most importantly Begomoviruses (family *Geminiviridae*), the largest genus of plant infecting viruses that causes enormous economic losses worldwide (Czosnek et al., 2017; Kanakala and Ghanim, 2018). The transmission of many plant viruses by *B. tabaci* and its ability to develop resistance against many pesticides makes it one of the most devastating insect pests known in agriculture (Skaljac et al., 2017).

The interaction between viruses and plant proteins that have roles in the infection process were previously studied to some extend (Hanley-Bowdoin et al., 2013). However, very little is known about insect proteins that participate in the transmission of plant viruses by insect vectors, especially those that have roles in circulative transmission, most importantly *B. tabaci*-transmitted begomviruses (Kanakala and Ghanim, 2016a), and the identification of such proteins and verifying their roles in the transmission remain a major challenge. The most studied system in this regards is the circulative transmission of *Tomato yellow leaf curl virus* from Israel (TYLCV-IL) by *B. tabaci* (Ghanim, 2014). Several *B. tabaci* proteins have been identified and verified by various methods and the results have confirmed the role of these proteins in the circulative transmission of TYLCV. Those proteins include two heat shock proteins, a GroEL chaperone encoded by endosymbiotic bacteria of *B. tabaci*, and recently a cyclophilin B (CypB) protein (Ghanim, 2014; Rosen et al., 2015; Kanakala and Ghanim, 2016a, 2018). Those proteins were shown to interact with TYLCV and their inhibition influenced the persistence, circulation, and transmission of the virus. The interaction between the candidate proteins and their specific antibodies could interfere with the virus–protein interaction and thus influence the virus transmission and persistence in the insect. For example, it was demonstrated that feeding *B. tabaci* with specific anti-CypB, anti-Hsp70 and anti-GroEL specific antibodies caused significant effects on TYLCV persistence and transmission, and its localization in the midgut (Kanakala and Ghanim, 2016a).

RNA silencing by introducing gene-specific dsRNA molecules is another approach that have been widely used to study insect development and virus–vector interactions in many organisms including insects (Kanakala and Ghanim, 2016a). For example, several delivery methods were used for introducing dsRNAs into *B. tabaci*, including injection for silencing genes expressed in the midgut and salivary glands and those that have a role in egg development (Ghanim et al., 2007). Artificial feeding through membranes was used for the silencing of several candidate genes such as an actin ortholog, ADP/ATP translocase, alpha-tubulin, ribosomal protein L9 (*RPL9*), and Vacuolar-type ATP*ase* A subunit, which caused varying levels of mortality (Upadhyay et al., 2011). Similar feeding methods were used for knocking down the cytochrome P450 monooxygenase *CYP6M1* gene, which showed increased mortality and influenced the detoxification ability of imidacloprid and nicotine in both MEAM1 and MED *B. tabaci* species (Li et al., 2015). Recently, the expression of dsRNA of whitefly genes inside the entomopathogenic fungi, *Isaria fumosorosea* (Chen et al., 2015) and endosymbiotic bacteria (Whitten et al., 2016) were successfully used to induce silencing of target genes in the insect host. Further recent studies demonstrated that whiteflies feeding on transgenic tobacco plants expressing dsRNA against the *v-ATPase A* gene (Thakur et al., 2014) and the osmoregulators *aquaporin* (*AQP*) and *alpha glucosidase*, (*AGLU*) (Raza et al., 2016), significantly reduced the expression level of targeted genes in *B. tabaci* and caused high mortality rates after feeding. So far, none of the gene silencing cases reported from *B. tabaci* were related to virus transmission.

Cyclophilins (Cyps) are a large family of cellular proteins with prolyl isomerase activity that have many molecular roles as chaperons and as signaling molecules (Wang and Heitman, 2005). Recently, CypB from the cereal aphid *Schizaphis graminum* was shown to interact with *Cereal yellow dwarf virus* (CYDV-RPV), and its expression was correlated with higher ability of some aphid biotypes to transmit CYDV-RPV (Tamborindeguy et al., 2013). We have recently shown that CypB interacts with TYLCV in *B. tabaci*, while inhibiting this protein with a specific inhibitor influenced the virus stability in the gut, and its transmission by the insect (Kanakala and Ghanim, 2016a). In a similar study, we demonstrated that *B. tabaci* Hsp70 interacted with TYLCV in the gut. But unlike CypB, feeding whiteflies with anti-Hsp70 specific antibodies resulted in higher transmission rates of TYLCV. This result suggested that Hsp70 had a protective role against the virus, which has been shown to induce various negative effects to the insect (Götz et al., 2012; Ghanim, 2014). Heat shock proteins (Hsps) belong to a multifunctional chaperon families that are upregulated by cells under stress, such as viral and bacterial infections, and play a crucial role in various cellular processes (Li and Srivastava, 2004). Since both CypB and Hsp70 were implicated in the transmission of TYLCV by *B. tabaci*, both proteins were selected as candidates for plant-mediated whitefly gene silencing using the *Tobacco rattle virus* (TRV) system and testing the effect of their knock-down on TYLCV transmission and whitefly development. Here we show that silencing both *CypB* and *Hsp70* genes in *B. tabaci* resulted in impaired nymphal, adult and endosymbiont development, as well as reduced fecundity and high mortality in the progeny of *B. tabaci* adults, which were exposed to TRV-silencing plants. Silencing both genes further resulted in reduced TYLCV transmission ability and confirmed the role of both genes in TYLCV–whitefly interactions.

## Materials and Methods

### Insects, Virus Source, and Plants

*B. tabaci* B biotype (MEAM1) populations used in this study were reared on cotton seedlings (*Gossypium hirsutum* L. cv. pima) maintained inside insect-proof cages within growth chambers at 26°C, 60% relative humidity and a 14 h light/10 h dark photoperiod. One-week-old adult whiteflies were used for the feeding experiments. Tomato seedlings (*Solanum lycopersicum* cv. Avigail) plants were agroinoculated with partial tandem repeats (PTR) construct of TYLCV DNA A (Navot et al., 1991), and new infected plants were generated by whitefly-mediated inoculation which were used as the virus source for the experiments in this study. The purity of *B. tabaci* B biotype was confirmed using microsatellite Bem 23 primers and cytochrome C oxidase (COI) gene sequencing (De Barro et al., 2003). Presence of TYLCV in insects and plants was confirmed using the V1 and C473 primers (Ghanim et al., 1998) (Supplementary Table S1). Tomato plants at their three to five true leaf stages were used for virus transmission experiments.

### Generation of Recombinant *Tobacco Rattle Virus* (TRV) Vectors

Total RNA of *B. tabaci* and tomato plants were extracted using TriZol reagent (Invitrogen, United States) and the first strand cDNA was synthesized using Verso cDNA kit (Thermo scientific, Fermentas) following the manufacturer’s instructions. Fragments of *B. tabaci* *cypB* and *hsp70* genes were amplified from the cDNA with Bt *CypB* F/R and Bt *Hsp70* F/R primers pairs, respectively (Supplementary Table S1). For silencing tomato *CypB* gene as a control, a fragment of 302 base pairs was amplified using tomato cDNA as a template with the primers Tom *CypB* F/R primers pairs. All the fragments were T/A cloned into the pGMET vector (Promega), excised using *Eco*R1 and *Bam*H1 and ligated to *Tobacco rattle virus* 2 (TRV 2) using the same restriction enzymes. The silencing TRV vectors including TRV1 and TRV2 were kindly provided by Prof. Henryk Czosnek from the Hebrew University of Jerusalem. The tomato gene *Hsp90* was chosen as positive control to evaluate the silencing effect mediated by TRV, while TRV1 and TRV2 alone were used a negative control.

### Agroinoculations and Virus Detection

The TRV1 and four TRV2 constructs: TRV2-Bt*CypB*, TRV2-Bt*Hsp70*, TRV2-Tom*CypB* and TRV2-Tom*Hsp90* were separately transformed into *Agrobacterium tumefaciens* C58 strain. The transformed *A. tumefaciens* cells were grown at 28°C in Luria–Bertani (LB) medium containing kanamycin and rifampicin (50 μg/mL each) for 24 h. pellets were resuspended in 10 mM MgCl_2_, 10mM MES (2-(*N*-Morpholino) ethanesulfonic acid) and 120 mM acetosyringone) and kept at 25°C for 3 h. The suspensions of *A. tumefaciens* cells were diluted to OD_600_ of 0.8. Each recombinant TRV2 virus construct was mixed with TRV1 virus in equal concentrations and inoculations of 2-week-old 30 tomato seedlings was performed by agro-infiltration (Senthil-Kumar and Mysore, 2014). Agroinoculated plants were maintained under 25 ± 2°C in growth rooms and phenotypic changes were recorded periodically. After 7 days post inoculation (dpi), the movement of TRV1 and four TRV2 constructs in tomato plants was detected by reverse transcription PCR (RT-PCR) analysis with vector and target gene specific primers (Supplementary Table S1). PCR positive plants were later transferred to insect-proof cages for insect bioassays.

### *B. tabaci* Bioassays With TRV-Inoculated Plants

*B. tabaci* RNAi bioassays were performed with 2-week-old plants after agroinoculation with the respective construct. Two plants from each combination, TRV1+TRV2-Bt*CypB*, TRV1+TRV2-Bt*Hsp70*, and TRV1+TRV2 as control were transferred to individual insect-proof cages. For feeding experiments, newly hatched whiteflies from cotton plants were used as non-viruliferous insects. Other newly hatched whiteflies were transferred to TYLCV-infected tomato plants for 48 h acquisition access period and those were used as viruliferous insects. For each replicate around 300 viruliferous or non-viruliferous whiteflies were transferred to each group of plants in insect-proof cages. Quantification of target gene mRNAs was carried out from adults that were fed on the plants for 24 h, 72 h, and 168 h. After 1 week, additional insects were removed from the plants for analyzing the progeny. Plants were monitored every day for counting egg hatch and nymphal development. First generation progeny (nymphs) on all groups of TRV plants were collected and used for quantitative Real Time PCR (qRT-PCR), fluorescent *in situ* hybridization (FISH) assays and for light microscopy.

*B. tabaci* mortality assay was performed using 15 adult female whiteflies which were fed on all plant groups using clip-on-leaf cages. After 48 h, adults were removed from the leaf cage, and the exposed leaf area was marked with marker and eggs were counted. Plants were shifted to insect free cages and were monitored every 2 days for mortality and fecundity tests. Egg mortality was assessed after 8–10 days by counting number of viable nymphs. Eggs that had failed to hatch or dried out, or nymphs that had died on hatching, were scored as dead. Plants were maintained to collect first generated whiteflies. About 15 first generation female whiteflies were collected from the above assay and were fed on new tomato plants leaves to test fecundity. The experiments were repeated three times and five biological replicates were included in each experiment.

### DNA and RNA Extractions, RT-PCR, and qRT-PCR

For total RNA extractions, adult whiteflies (30 whiteflies/ replicate) and nymphs (50 nymphs/replicate) were collected at each time point from tomato plants and RNA was isolated using TRIzol reagent (Sigma-Aldrich) then was treated with DN*ase*I according to the manufactures recommendations (Thermo Scientific). RNA (100 ng) was used as a template for cDNA synthesis in 25 μl reaction mixtures by using Verso cDNA kit (Thermo Scientific, Fermentas). For DNA isolation, a pool of 50 nymphs per replicate was isolated using CTAB (cetyltrimethylammonium bromide) method (Shahjahan et al., 1995). The same DNA was used for the screening all three symbionts *Portiera*, *Hamiltonella*, and *Rickettsia* using 16S rDNA specific primers (Supplementary Table S1). PCRs were carried out as previously described (Chiel et al., 2007). For qPCR, 3 ng of DNA was used in triplicates for each DNA sample. Five to six biological replicates and three technical ones for each biological replicates were used in all experiments.

The plant DNA was extracted from 100 mg of tomato leaf tissue, as described previously (Kanakala and Ghanim, 2016a). For qPCR, 50 ng of DNA was used in triplicates for each DNA sample. RNA was extracted from 100 mg of leaf tissue using TRIzol (Sigma-Aldrich), and the cDNA synthesis was performed as instructed by the manufacturer using Verso cDNA kit (Thermo Scientific, Fermentas). To ensure the validity of the data, cDNA was used for qRT-PCR in triplicate for each cDNA sample.

Target mRNAs and TYLCV in adult whiteflies and bacterial density in nymphs were quantified by qRT-PCR using Fast SYBR Green Rox mix (Thermo Scientific) using Rotor-Gene 6000 machine (Corbett Robotics Pty Ltd., Brisbane, QLD, Australia) and the accompanying software for qPCR data processing and analysis. The cycling conditions were: 15 min activation at 95°C, 45 cycles of 10 s at 95°C, 20 s at 60°C and 25 s at 72°C. Tomato *β-actin* and *B. tabaci* *β-actin* (Sinisterra et al., 2005), α-*tubulin/BT-C02/BT-E06* (Mahadav et al., 2009) genes were used as internal controls for normalization after validation. The primers used for tomato and *B. tabaci* and symbionts are given in Supplementary Table S1. All assays were carried out in triplicates in each of three biologically independent experiments.

### Fluorescence *in situ* Hybridization (FISH)

FISH was performed as previously described (Gottlieb et al., 2006). Briefly, nymphs were fixed overnight in Carnoy’s fixative (chloroform:ethanol:glacial acetic acid, 6:3:1, vol/vol). After fixation, the samples were decolorized in 6% H_2_O_2_ in ethanol for 2 h and then hybridized overnight in hybridization buffer (20 mM Tris–HCl pH 8.0, 0.9 M NaCl, 0.01% wt/vol sodium dodecyl sulfate, 30% vol/vol formamide) containing 10 pmol fluorescent probe/mL. For specific targeting of *Portiera* *Rickettsia* and *Hamiltonella*, the probes BTP1-Cy3 (5′-Cy3-TGTCAGTGTCAGCCCAGAAG-3′) (Gottlieb et al., 2006), Rb1-Cy5 (5′-Cy5-TCCACGTCGCCGTCTTGC-3′) (Gottlieb et al., 2006) and BTH-Cy5 (5′-CCAGATTCCCAGACTTTACTCA-3′) (Gottlieb et al., 2008), respectively, were used. Nymphs were stained with DAPI (4′,6′-diamidino-2-phenylindole) at 0.1 mg mL^-1^. The stained samples were mounted whole in hybridization buffer and viewed under an IX81 Olympus FluoView500 confocal laser-scanning microscope. For each treatment, 20–30 nymphs were viewed. Optical confocal sections (100-μm thick) were sometimes prepared from each specimen for better visualization of the signal.

### Transmission of TYLCV Following Feeding on Tomato Plants Expressing *CypB* and *hsp70* dsRNA

To assess the implication of CypB and Hsp70 in the transmission of TYLCV, 1-week-old *B. tabaci* adults were given acquisition on TRV-Bt*CypB* and TRV-Bt*hsp70* inoculated plants for a week. The insects were then transferred to TYLCV-infected tomato plants for additional 48 h acquisition access period and subsequently single whiteflies were transferred to tomato plants in their three-leaf stage for 7 days. Whiteflies fed on TRV alone and TYLCV-infected plants for 48 h served as a controls. Tomato plants were grown in a potting mix in 1.5L pots under artificial light and maintained inside insect-free greenhouse under controlled temperature as detailed above. The whiteflies that were incubated with the plants were tested for TYLCV acquisition. The plants were monitored for the development of disease symptoms after 28 days post inoculation. DNA was extracted from symptomatic and non-symptomatic tomato plants and subjected to PCR for detecting TYLCV with specific coat protein (CP) primers V61 and V473 (Ghanim et al., 1998). The experiments were triplicated with 20 plants for each replicate.

### Statistical Analysis

**FIGURE 1:**
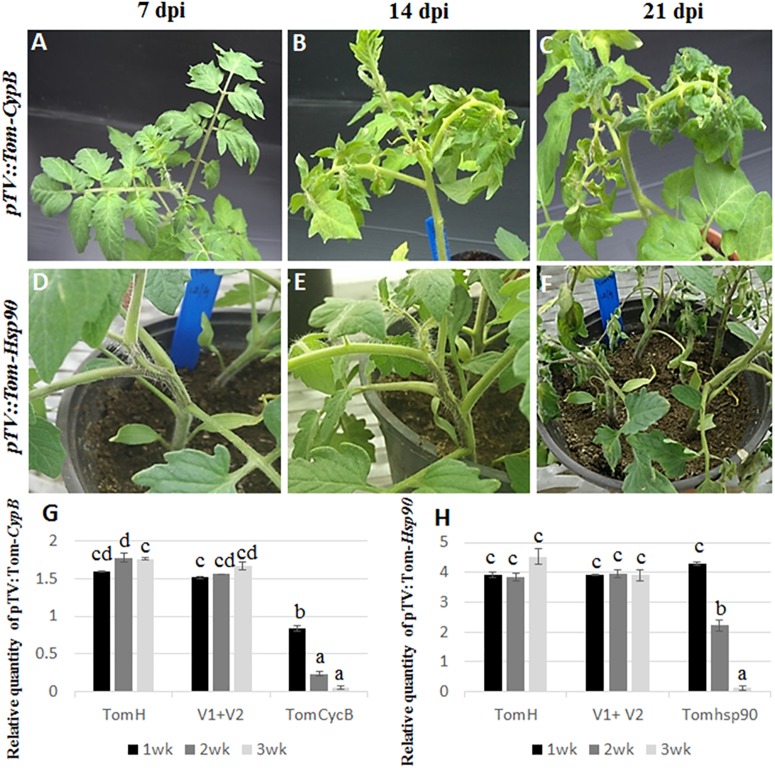
Silencing of tomato *Cyp B* and *Hsp 90* genes. Silencing of *CypB* **(A–C)** induces leaf petiole rolling and leaf crinkling after 14 and 21 days post infection. Silencing of tomato *Hsp90* gene **(D–F)** induces cell death after 21 days post infection. **(G)** qRT-PCR of tomato *CypB* **(G)** and *Hsp90* **(H)** genes in three TRV lines at 7, 14, and 21 days post infection showing effective silencing of both genes compared to the controls. TomH is a non-agroinfiltrated tomato control. V1+V1 is a control agroinfiltrated with both control plasmids. TomCycB and TomHsp90 are plant agroinfiltrated with the respective gene for silencing (CycB or Hsp90).

**FIGURE 2:**
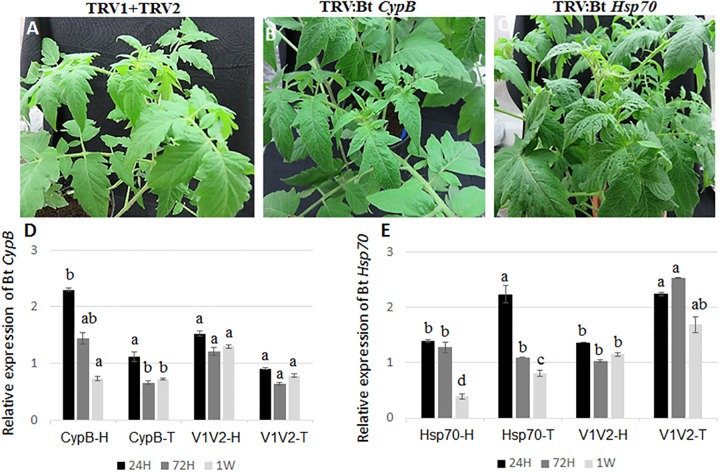
Development of tomato plants after agroinfiltration with **(A)** TRV1+TRV2 (control), **(B)** TRV::Bt *CypB*, **(C)** TRV::Bt *Hsp70*. The pictures shown were takes 4 weeks post agroinfiltration. **(D)** Relative expression of Bt *CypB* in healthy tomato (CypB-H), TYLCV-infected tomato (CypB-T) and in control plants infected (V1V2-T) and uninfected (V1V2-H) with TYLCV after agroinfiltration with both TRV constructs. **(E)** Relative expression of Bt *Hsp70* in healthy tomato (Hsp70-H), TYLCV-infected tomato (Hsp70-T) and in control plants infected (V1V2-T) and uninfected (V1V2-H) with TYLCV after agroinfiltration with both TRV constructs. Bt, *Bemisia tabaci.*

**FIGURE 3:**
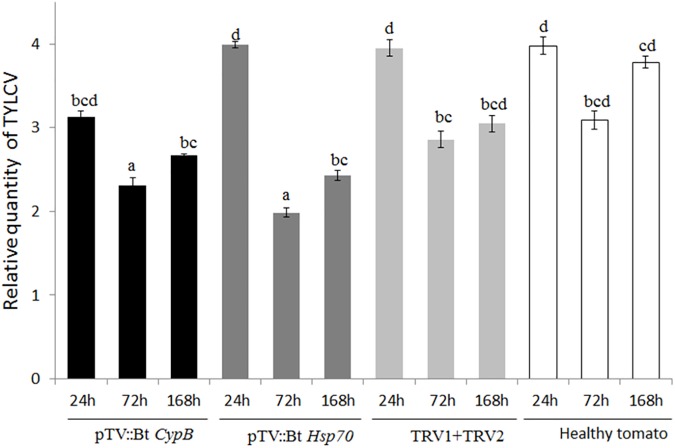
Quantification of TYLCV levels in *B. tabaci* adults feeding on plants expressing dsRNA against *Cyp* B and *Hsp70* after 24 h, 72 h, and 1 week (168 h), compared to mock inoculated plants and plants inoculated with both TRV1 and TRV2 alone as a control.

A one-way analysis of variance using Tukey’s HSD adjustment test for multiple comparisons at a family-wise error rate of 5% was used for comparing all possible pairs of treatments. The connected letters in Figure 1–3, 5–7 provide a graphical summary: treatments not connected by the same letter are significantly different (i.e., Tukey adjusted *p*-value < 0.05). Error bars on the barplots are standard error of mean.

## Results

### Testing the System With TRV-Mediated Silencing of Tomato Genes

Tomato plants transiently expressing dsRNA of *B. tabaci* and tomato target genes were generated by agroinfiltration. After a week, total RNA from agroinfiltrated plants was subjected to reverse transcription-PCR to amplify the target genes and TRV using specific primers. Amplification of the desired bands and virus confirmed the systemic movement of the TRV and target gene mRNA required for inducing siRNA production. First, the working system was tested for the ability to silence tomato genes. Plants agroinfiltrated with pTV::Tom-*CypB* construct, made for silencing the tomato *CypB* gene caused significant leaf petiole rolling and leaf crinkling, while the plants remained retarded until 8 weeks after infiltration, and those symptoms extended until death (Figure 1A–C). The expression of the tomato *CypB* gene in those agroinfiltrated plants was monitored using qRT-PCR analysis, and the results showed significant decrease in the transcript levels at 7 dpi which were further reduced to significantly lower levels by 14 and 21 dpi (Figure 1G). The expression of the tomato *CypB* gene, however, remained stable in the control plants which were not infiltrated or which were infiltrated with control TRV1 and TRV2 constructs (Figure 1G). Tomato plants were also co-infiltrated with pTV::Tom-*Hsp90* construct targeted to the endogenous heat shock protein 90 gene of tomato (Moshe et al., 2016) as an additional positive control. The virus propagation was visualized in stems 1 week after agroinfiltration and the stem became necrotic and plants died 24 days post infiltration as previously described (Figure 1D–F) (Moshe et al., 2016). The levels of the tomato *Hsp90* transcripts over time showed gradual decrease over 21 dpi (Figure 1H), compared to the controls in which the levels of the transcript remained stable over the course of the experiment. Altogether, the above results confirmed the ability of the TRV system to knock down the expression of tomato target genes to very low levels and cause significant phenotypes in the plant that resulted in death.

### TRV-Mediated Silencing of *B. tabaci* Genes and the Effect on Virus Levels

Selected regions of the cDNA of *CypB* and *Hsp70* genes were used for dsRNA expression in tomato plants (Supplementary Figure S1). The *CypB* gene codes for a Cyclophilin B gene (accession number KX268377) and the RNAi construct was designed to target the conversed two β sheets and α-helices from amino acids 1–77 which are conserved in *B. tabaci* compared to other arthropods (Kanakala and Ghanim, 2016a). This gene family exhibits a peptidylpropyl *cis*–*trans* isomerases activity (PPIases) and highly conserved Cyclosporin (CsA)-binding residues. On the other hand, the *Hsp70* gene codes for heat shock protein 70, which are important for protein folding and responding to a variety of stresses in the cell (Morano, 2007). *Hsp70*-RNAi construct was designed to be specific to nucleotides unique to the conserved protein domain family NLPC_P60. *B. tabaci CypB* and *Hsp70* are sufficiently different from the tomato *CypB* and *Hsp70* genes, thus they were expected to be suitable for knockdown and obtaining specific results for *B. tabaci* genes. The CypB and Hsp70 amino acid phylogenetic analysis of *B. tabaci* and tomato genes are shown in Supplementary Figure S2.

To evaluate the ability of TRV-infected plants to express *B. tabaci* *CypB* and *Hsp70* dsRNA and induce silencing in whiteflies, tomato plants were agroinfiltrated with the TRV::Bt *CypB* and TRV::Bt *Hsp70* constructs separately. Agroinfiltration with TRV::Bt *CypB* (Figure 2B) and TRV::Bt *Hsp70* (Figure 2C) did not affect tomato plant development, and no major visible morphological differences were observed between those plants and control plants (Figure 2A). Next, the effect of the expressed dsRNA of *B. tabaci* *CypB* and *Hsp70* genes in tomato using TRV was tested on the whitefly development, gene expression and TYLCV transmission by feeding assays. Following feeding on pTV::Bt *CypB*, pTV::Bt *Hsp70*, TRV alone and mock inoculated plant leaves at three time intervals (24 h, 72 h, and 1 week), total RNA was extracted from both viruliferous and non-viruliferous whiteflies feeding on those plants and used in qRT-PCR. For Bt *CypB*, a gradual and significant down-regulation was observed from 24 h up to 1 week in non-viruliferous whiteflies (Figure 2D), while in viruliferous whiteflies a significant decrease in the expression was observed only at 72 h and 1 week compared to the levels at 24 h (Figure 2D). After *Hsp70* silencing, significant decrease in the expression was observed after 1 week in non-viruliferous whiteflies, while a gradual significant decrease was observed at 72 h and 1 week after the start of the feeding in viruliferous whiteflies (Figure 2E). The levels of the transcripts of both *CypB* and *Hsp70* genes remained stable in viruliferous and non-viruliferous whiteflies fed on the TRV1+TRV2 control plants (Figure 2D,E). Next, the virus levels in these experiments were also measured at the same time points where gene expression was measured. The results showed that the most significant effect on virus levels was observed in viruliferous whiteflies feeding on TRV::Bt *Hsp70* dsRNA expressing plants after 72 h post whitefly release, while in the rest of the treatments the virus levels did not significantly decrease at the same magnitude, possibly due to virus degradation over time (Figure 3).

### TRV-Mediated Silencing of *CypB* and *Hsp70* Induce Morphological Abnormalities in First Generation

**FIGURE 4:**
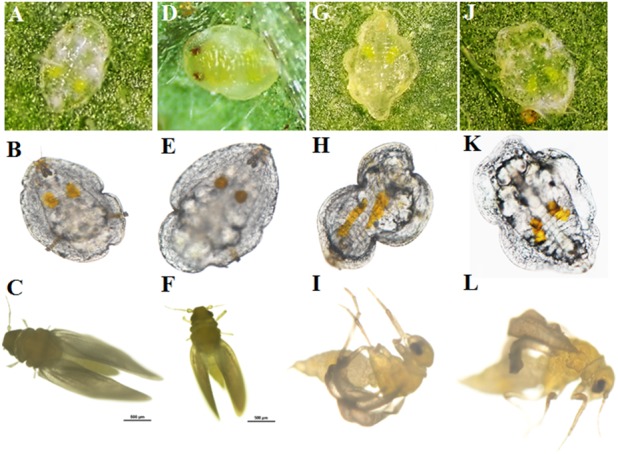
Phenotypes of nymphs and adults of *B. tabaci* first generation after feeding on plants expressing dsRNA against *CypB* and *Hsp70*. **(A–C)** Nymphs and adult offspring of whiteflies that fed on healthy tomato leaves. **(D–F)** Nymphs and adult offspring of whiteflies that fed on TRV-agroinfiltrated tomato. **(G–I)** Nymphs and adult offspring of whiteflies that fed on pTV::Bt *CypB* dsRNA expressing tomato leaves. **(J–L)** Nymphs and adult offspring of whiteflies that fed on pTV::Bt *Hsp70* dsRNA expressing tomato leaves.

In the next experiments, we monitored whether the effects of gene silencing induced in the adults that fed on the dsRNA-expressing TRV plants, can be passed to the next generation and induce silencing and possibly phenotypic changes. Tomato plants agroinfiltrated with the pTV::Bt *CypB*, pTV::Bt *Hsp70*, TRV alone and mock inoculated plants were used for feeding adult whiteflies for 48 h after which they were transferred to cotton plants for egg lay. This transfer to cotton was performed to avoid any dsRNA acquisition by the next generation individuals from plants, and any observed effects would be attributed to dsRNA acquired from the previous generation. After a week, microscopic observations on the cotton leaves surfaces showed no difference in hatching between treatments and control insects. In all the agroinfiltrated groups, crawlers were observed over the leaf surface in search for a suitable settling site. Progeny collected from the pTV::Bt *CypB*, pTV::Bt *Hsp70* showed phenotypic changes in second and third instar stages compared to TRV alone and mock infiltrated (Figure 4). Over all, different phenotypes were observed between nymphs fed on Bt *CypB* and Bt *Hsp70* dsRNA-expressing plants. In the control insects, all instars looked oval in shape and flattened dorso-ventrally (Figure 4A,B,D,E). In case of nymphs feeding on *CypB* dsRNA-expressing plants, they appeared irregular in shape and eventually dried and fall off the leaves (Figure 4G,H). Interestingly, great effect on development at red-eyed nymph stage was noted and the red eyes were not observed as in normal development. Nymphs fed on pTV::Bt *Hsp70* plants showed different phenotypic changes and the nymphs became flat and thin in size, edges of the nymph became more transparent and gradually dissolved on the leaf surface and dried (Figure 4J,K). We further examined the morphology of adults in the first generation of insects that have fed on dsRNA-expressing plants. In both TRV *CypB*- and TRV *Hsp70* dsRNA-expressing plants on which the first generation was feeding, the adults exhibited curled winged morphology and lighter color of the body (Figure 4I,L), compared to control whiteflies that fed on control plants (Figure 4C,F). These whiteflies could not fly because of the wing morphology, hardly moved on the leaf, and died within 2–3 days without laying any eggs.

**FIGURE 5:**
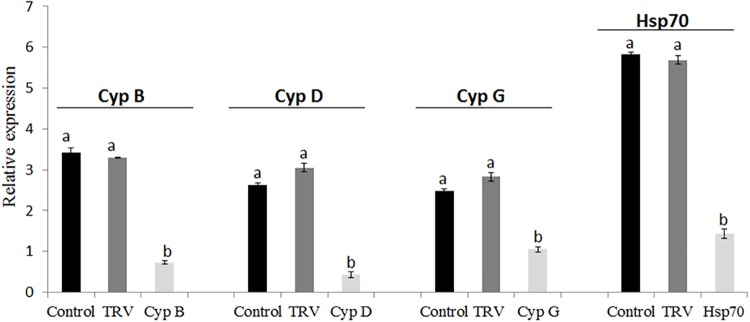
Quantification of *B. tabaci* *Cyp* genes (*CypB*, *CypD*, and *CypG*) and *Hsp70* transcripts in next generation nymphs feeding on healthy and TRV control plants, and on pTV::Bt *CypB* and pTV::Bt *Hsp70* dsRNA-expressing tomato plants.

In these experiments, we quantified mRNAs levels in *B. tabaci* nymphs of the first generation that developed on the TRV-*CypB*, TRV-*Hsp70*, TRV alone and mock inoculated tomato plants using qRT-PCR in pools of second and third nymphal stages. The results showed dramatic suppression of the endogenous *CypB* and *Hsp70* mRNAs (Figure 5). Quantification of the amount of *CypB* and *Hsp70* genes were normalized against constitutively expressed *B. tabaci* α-tubulin levels. We observed ∼3 times depletion of C*ypB* in nymphs when fed on TRV-*CypB* plants. Simultaneously, the expression of *Cyp* D and G genes, which were previously studied by us, was tested and found to be reduced by ∼2.5 and 1.5 times, respectively (Figure 5). These results imply that knockdown of *CypB* could affect other *Cyp* genes in the whitefly. In the case of *Hsp70*, mRNA levels were reduced by ∼4 times when fed on TRV-*Hsp70*, compared to the controls.

### Effect of TRV-Mediated Silencing of *CypB* and *Hsp70* on Mortality, Fecundity, and Virus Transmission

The effect of silencing *CypB* and *Hsp70* on adult whitefly mortality and fecundity were examined. Experiments were performed over a period of 2 weeks in which adults were given access to TRV-silencing plants for both genes and the cumulative mortality and fecundity (number of laid eggs) were recorded. Surprisingly, we obtained very high mortality levels that reached 81.8% after feeding on TRV-*CypB* plants, and 85.6% when the adults were fed on TRV-*Hsp70* plants, compared to the TRV and mock control plants where the mortality reached 20% and 16%, respectively.

**FIGURE 6:**
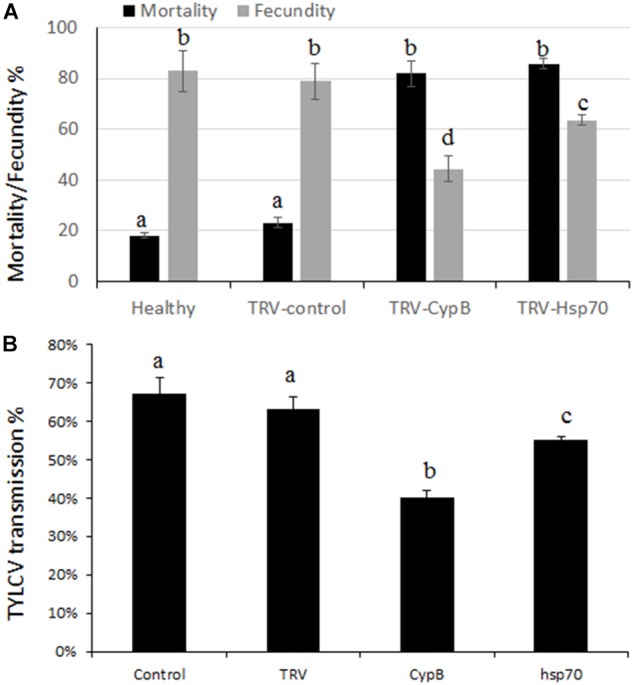
Effect of TRV-silencing *CypB* and *Hsp70* on mortality and fecundity **(A)** and on TYLCV transmission **(B)**.

Females that did not die following feeding on the TRV-silencing plants were recovered from all treatments. Ten females for each replicate were used for egg lay over a course of 2 weeks. The results showed that females feeding on TRV-*CypB* or TRV-*Hsp70* plants laid 38.6% and 19.4% less eggs, respectively, compared to the control TRV and mock plants (Figure 6).

Some of the recovered females after feeding on silencing plants for both genes were used for virus transmission experiments. Adult MEAM1 females fed on TRV-*CypB* and TRV-*Hsp70* expressing plants for a week or on TRV alone and mock agroinfiltrated plants as control, were caged with TYLCV-infected plants for a 48 h acquisition access period. Subsequently, single whiteflies were transferred to tomato plants in their three-leaf stage for 7 days inoculation access period. In the TRV-*CypB* and TRV-*Hsp70* infected plants, 40% and 55% transmission rates were obtained on average, compared to ∼67% transmission rate when the whiteflies were fed on control plants (Figure 6).

### Silencing *CypB* and *Hsp70* Induce Endosymbiont Displacement and Reduce *Rickettsia* Amounts

**FIGURE 7:**
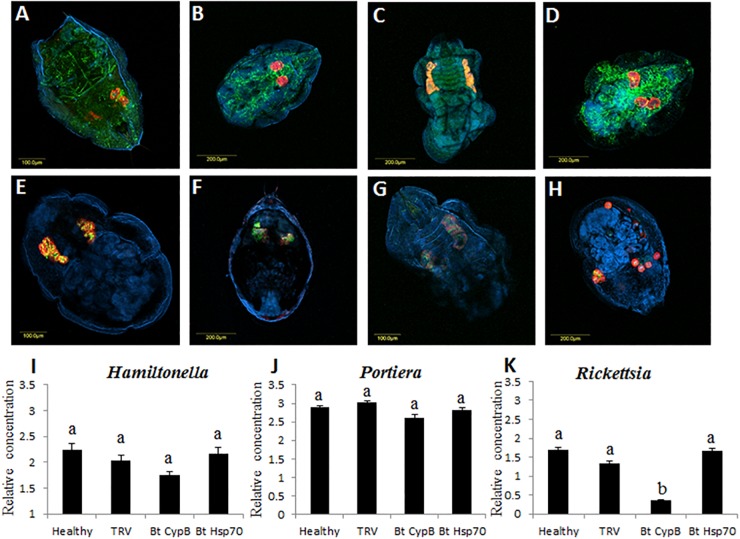
Effect of *CypB* and *Hsp70* silencing on localization and densities of bacterial symbionts in *B. tabaci.* Localization of *Portiera* (red) and *Rickettsia* (green) is shown in **(A)** and localization of *Portiera* (red) and *Hamiltonella* (green) is shown in **(E)**, in both cases the insects were fed on healthy control plants. **(B,F)** are the same treatments and the insects were fed on TRV control plants. **(C,G)** the insects were fed on TRV-*CypB* plants in which *CypB* was silenced and in **(D,H)** the insects were fed on TRV-*Hsp70* plants in which *Hsp70* was silenced. In **(I–K)** quantification of the symbionts *Hamiltonella, Portiera*, and *Rickettsia*, respectively, using qPCR in healthy and TRV control plants and in TRV-*CypB* and TRV-*Hsp70* plants in which both the investigated genes *CypB* and *Hsp70* were silenced.

*B. tabaci* is associated with bacterial endosymbionts that contribute to its successful biology and interactions with the environment (Gottlieb et al., 2008; Gueguen et al., 2010; Skaljac et al., 2017). *Portiera* is the primary endosymbiont while there were seven additional secondary symbionts reported from *B. tabaci* cryptic species around the world. The amounts and density of these different symbionts can be influenced from internal genetic factors in the whiteflies, as well as external environmental factors, which in turn can influence the whitefly biology (Brumin et al., 2011). In the current study, we tested whether the location and densities of the three endosymbionts *Portiera, Rickettsia*, and *Hamiltonella* that infect the MEAM1 species that we used in this study are influenced following the silencing of *CypB* and *Hsp70.* As seen in Figure 7, the location of all tested symbionts did not change between the control (Figure 7A,B,E,F), and insects that where fed on silencing plants (Figure 7C,D,G,H). *Portiera* and *Hamiltonella* colocalized inside the bacteriome cells, while *Rickettsia* localized outside the those cells, and occupied outside organs in the body cavity as previously reported (Gottlieb et al., 2008; Brumin et al., 2012). However, the most notable difference between the control and silenced insects was the shape and structure of the bacteriome cells. In the control insects, the cells looked intact and appeared as one structure, while in the insects in which *CypB* was silenced, the bacteriome looked elongated and the cells partially disintegrated (Figure 7C,G). In the insects in which *Hsp70* was silenced, the phenotypes were even more severe where the bacteriome cells looked completely disintegrated, and the cells appeared all over the body when *Portiera* and *Hamiltonella* were localized (Figure 7H). When the densities of the three symbionts were measured after silencing using qPCR, the amounts of *Portiera* and *Hamiltonella* did not significantly differ compared with the controls, however, the amounts of *Rickettsia* were significantly reduced when *CypB* was silenced, a result that could be observed in the FISH results (Figure 7C).

## Discussion

In this study, we attempted to develop an RNAi-based approach for controlling one of the most important insect pests known in agriculture, by targetting two proteins that have been previously implicated in TYLCV transmission by *B. tabaci.* Over the past decade and since RNA interference (RNAi) was discovered, this technology has been described as a promise to develop effective, friendly and inexpensive technologies for making crops resistant to insect pests (Baum et al., 2007; Mao et al., 2007). Indeed, hundreds of research results described the effective use of RNAi in causing mortality or reducing insect pest populations, including insect vectors by delivering dsRNA into the insects in various ways (Gordon and Waterhouse, 2007; Price and Gatehouse, 2008; Kumar et al., 2012, 2014; Wuriyanghan and Falk, 2013; Kanakala and Ghanim, 2016b). Here, we took advantage of the ability of *B. tabaci* to feed on tomato, to test the effectiveness of RNAi in causing mortality and reducing virus transmission. This was done by silencing cyclophilin B and Hsp70, two genes with verified role in TYLCV transmission. We also used the TRV silencing system since it was shown to be very effective and robust in tomato, and has been shown to produce stable dsRNA/siRNA against target gene in plants (Kanakala and Ghanim, 2016b).

For testing the functionality of this system, we silenced the tomato cyclophilin and Hsp90 genes. These experiments demonstrated the robustness of the silencing system, especially when *Hsp90* was silenced and the results obtained were as previously reported (Moshe et al., 2016). Next, *CypB* and *Hsp70* were targeted. Many recent studies demonstrated the ability to induce silencing in whiteflies, but none targeted genes involved in the insect–virus interactions or used the TRV plant-mediated silencing system (Ghanim et al., 2007; Thakur et al., 2014; Chen et al., 2015; Li et al., 2015; Raza et al., 2016; Whitten et al., 2016). TRV-mediated silencing of both *B. tabaci* *CypB* and *Hsp70* genes resulted in significant reduction in their expression (Figure 2). The silencing was consistent and reproducible 7 days post TRV inoculation and was observed in adults and nymphal stages feeding on the phloem of TRV plants expressing dsRNA for both genes (Figure 2, 1 week after silencing). After this successful silencing, subsequent experiments were designed to test the effectiveness of silencing *CypB* and *Hsp70* in viruliferous and non-viruliferous whitefly adults, and the results showed that the presence of the virus influenced the silencing potency, as well as the virus levels in the whitefly, confirming the role of these two genes in the whitefly–virus interactions.

Previous research studies in insects pests showed the effectiveness of RNAi in inducing phenotypic changes, as well as lethality, for example in planthoppers (Wu et al., 2012; Zhou et al., 2013; Xi et al., 2015), as we observed here. Although the experiments did not result in complete mortality and developmental arrest of all the nymphs on the leaves, highly significant number of nymphs did show phenotypic abnormalities, suggesting that the plant-mediated TRV silencing is an effective approach for dsRNA delivery by feeding in the plant phloem. The fact that silencing both *CypB* and *Hsp70* resulted in very high mortality rates suggests important roles that they play in the insect development, although the phenotypes obtained with both genes were not similar confirming their different roles in development. In the case of *Hsp70* the phenotypes appeared as drying of the nymphs and adults, suggesting internal factors that result in these drastic changes, while in the in the case of *CypB* more morphological changes in the nymphs were obtained. This may be attributed to the downregulation of the other *Cyp* genes (*CypD* and *CypG)*, which when silenced together with *CypB* may contribute to more drastic effects on interactions with other genes, misregulation in their cumulative roles in correct folding of proteins and more drastic phenotypes as we observed. *Hsp70* is expressed almost in every cell and organ and have unusual sequence conservation and functional roles (Boorstein et al., 1994; Karlin and Brocchieri, 1998). It belongs to a large multifunction chaperone family with expanded cellular processes including disaggregation or proteins, prevention of aggregation, correct folding pf proteins, direction of proteins to their targets including translocation across membranes and targeting proteins to specific cellular domains (Pratt et al., 1999; Pishvaee et al., 2000). Many proteins of this family have overlapping roles because of the many functions they have to fulfill, thus they are present ubiquitously in the cell. It also known that some plant viruses encode their own Hsp70 proteins which facilitate virus transport cell to cell in the plant (Alzhanova et al., 2001; Aoki et al., 2002), and have roles in the virus life cycle and replication (Wang et al., 2009). Similar to HSp70s, *Cyp* genes which belong to the peptidyl-prolyl isomerases proteins (PPIases or Cyps), have important roles in the proper folding, chaperone functions and as modulators for human virus replication (Frausto et al., 2013). They were shown to interact with the capsid protein of the human immunodeficiency virus type 1 (HIV-1) (Schaller et al., 2011) and influenza A virus M1 protein (Liu et al., 2009) and were shown to play a key role in the viral replication cycle. They were also shown to contribute to the transmission of B/CYDV circulation and transmission by aphids (Tamborindeguy et al., 2013), and were hypothesized to play a role in chaperoning these viruses to various membrane bound vesicles (Yang et al., 2008).

One of the major effects that we observed following the silencing of both *Hsp70* and *CypB* is the disruption of the endosymbiont location in the insect, however the amounts of the endosymbionts were not disrupted except in the case of *Rickettsia* that was reduced following the silencing of *CypB.* Silencing *Hsp70* showed a dramatic effect on the location of the bacteriosome cells and they appeared disintegrated from the main bacteriome and dispersed in the cytoplasm. These results are supported by previous research in which it was shown that *Rickettsia*, which is the only symbiont that localizes outside of bacteriosome cells is influenced by stress and environmental factors such as heat and virus infection (Brumin et al., 2012; Kliot et al., 2014; Su et al., 2014). However, the effect of silencing *Hsp70* on the integrity of the bacteriome is novel and have not been associated with silencing insect genes in the whitefly or other insects that harbor endosymbiotic bacteria.

Silencing of insect genes is known to influence insect fertility and fecundity as was shown in several reports (Thakur et al., 2014; Mysore et al., 2015; Upadhyay et al., 2016). In our study, we also observed decreased fertility and fecundity when both *B. tabaci* *CypB* and *Hsp70* were silenced. These effects correlated with decrease in number of eggs laid and the number of hatched eggs. Targeting *CypB* by silencing caused decrease in fecundity more than that of *Hsp70*. The decrease in fecundity and fertility correlated well with the levels of gene expression measured from whole body and nymphs preparations, suggesting that silencing those genes not only results in the mortality of the first generation adults, but also in the reduction of their next generation. Silencing *B. tabaci* vitellogenin (*vg*) gene has been previously shown to be a potent target for decreasing fecundity and inducing mortality (Upadhyay et al., 2016).

The results we obtained here, showed that the siRNA signal could be passed from the feeding females on the plants to its offspring, where it induced silencing in the next generation. Similar results demonstrated the passage of silencing signal between generations (Grentzinger et al., 2012; Abdellatef et al., 2015). Reduced *CypB* expression in next generation nymphs and the wing morphology we observed in the next generation adults as well as the phenotypic changes in the development of nymphs suggest an efficient mechanism by which the silencing signal is transferred. Whiteflies fed on *CypB* dsRNA-expressing plants died earlier than those fed on pTV::Bt *Hsp70*, TRV alone and mock inoculated plants, thus we were not able to test the effect on following generations.

The effect on virus transmission following silencing was an important goal of this study. The results showed that TYLCV transmission was reduced by 27% after *CypB* plant-mediated silencing, and less than that when *Hsp70* was silenced. These results are consistent with previous results in which inhibition of *CypB* resulted in decreased TYLCV transmission by *B. tabaci* by 43% (Kanakala and Ghanim, 2016a). As mentioned above, TYLCV transmission was reduced only by 12% after *Hsp70* silencing confirming previous results in which this protein was shown to pose an inhibitory role against TYLCV (Götz et al., 2012).

Taking these data together, our work demonstrated that targeting two previously identified genes in whitefly–virus interactions, *CypB* and *Hsp70*, could result in inhibiting the virus transmission and the quantity and location of endosymbionts in the insect. However, because these genes have many other functional roles in the cell, their silencing resulted in drastic phenotypic changes in nymphs and adults and in high mortality, and this silencing signal was heritable. Thus, those genes could serve as important targets for developing resistant plants against *B. tabaci*.

## Author Contributions

SuK and MG conceived and designed the study, analyzed the data, and wrote the manuscript. SuK, SvK, and GL conducted the experiments.

## Conflict of Interest Statement

The authors declare that the research was conducted in the absence of any commercial or financial relationships that could be construed as a potential conflict of interest.

## Supplementary Material

FIGURE S1 Partial sequences of the Bt *CypB*, Bt *Hsp70*, and Tomato *CypB*. **(A)** Bt *CypB* and **(B)** Bt *Hsp70* were amplified by PCR using gene-specific primers linked to *Eco*R1/*Bam*H1 restriction sites. **(C)** Tomato *CypB* was amplified by PCR using gene-specific primers linked to *EcoR1*/*BamH1* restriction sites. **(D)** The constructs of tobacco rattle virus vectors: the cDNA fragments were inserted into the multiple cloning site of pTRV2 vector to produce tobacco rattle virus constructs.

FIGURE S2 Phylogenetic tree of *B. tabaci* CypB **(A)** and hsp70 **(B)** and other arthropods, fungal species, *Solanum lycopersicum* and *H. sapiens*. Phylogenetic tree was generated using MEGA 6 (Tamura et al., 2013) with maximum likelihood method. Numbers next to the branches indicated bootstrap value of each internal branch in the phylogenetic tree nodes from 1,000 replicates.

TABLE S1 Primers used in this study for PCR and qRT-PCR analysis.
