# Supplementary figures and images for: Plant-Mediated Silencing of the Whitefly Bemisia tabaci Cyclophilin B and Heat Shock Protein 70 Impairs Insect Development and Virus Transmission
Source: Front Physiol. 2019 May 8;10:557. doi: 10.3389/fphys.2019.00557 (PMC6517521; doi:10.3389/fphys.2019.00557)

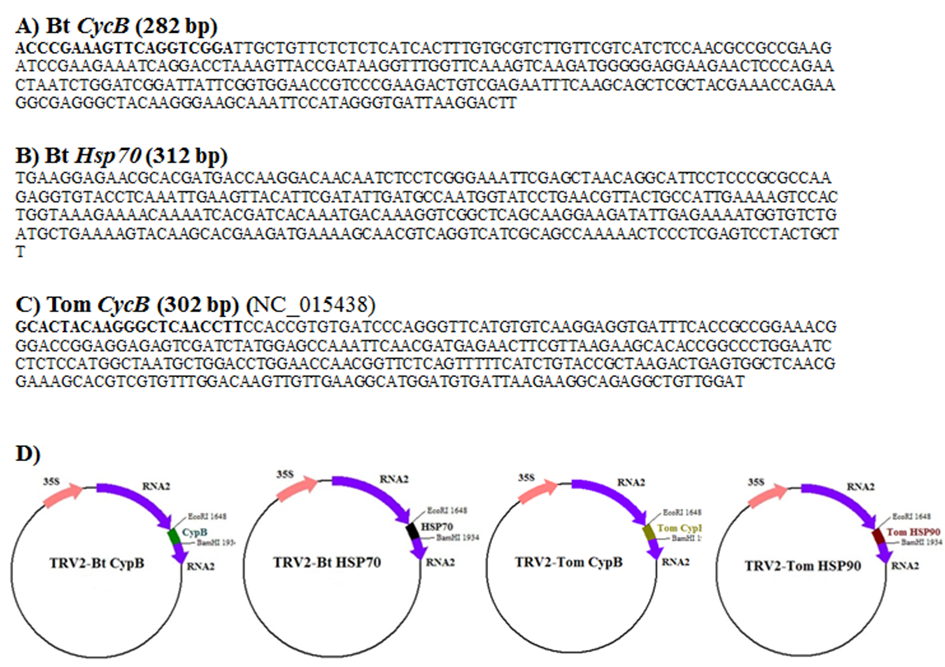

Supplement: FIGURE S1 — Partial sequences of the Bt CypB, Bt Hsp70, and Tomato CypB. (A) Bt CypB and (B) Bt Hsp70 were amplified by PCR using gene-specific primers linked to EcoR1/BamH1 restriction sites. (C) Tomato CypB was amplified by PCR using gene-specific primers linked to EcoR1/BamH1 restriction sites. (D) The constructs of tobacco rattle virus vectors: the cDNA fragments were inserted into the multiple cloning site of pTRV2 vector to produce tobacco rattle virus constructs. [file Image_1.TIF]

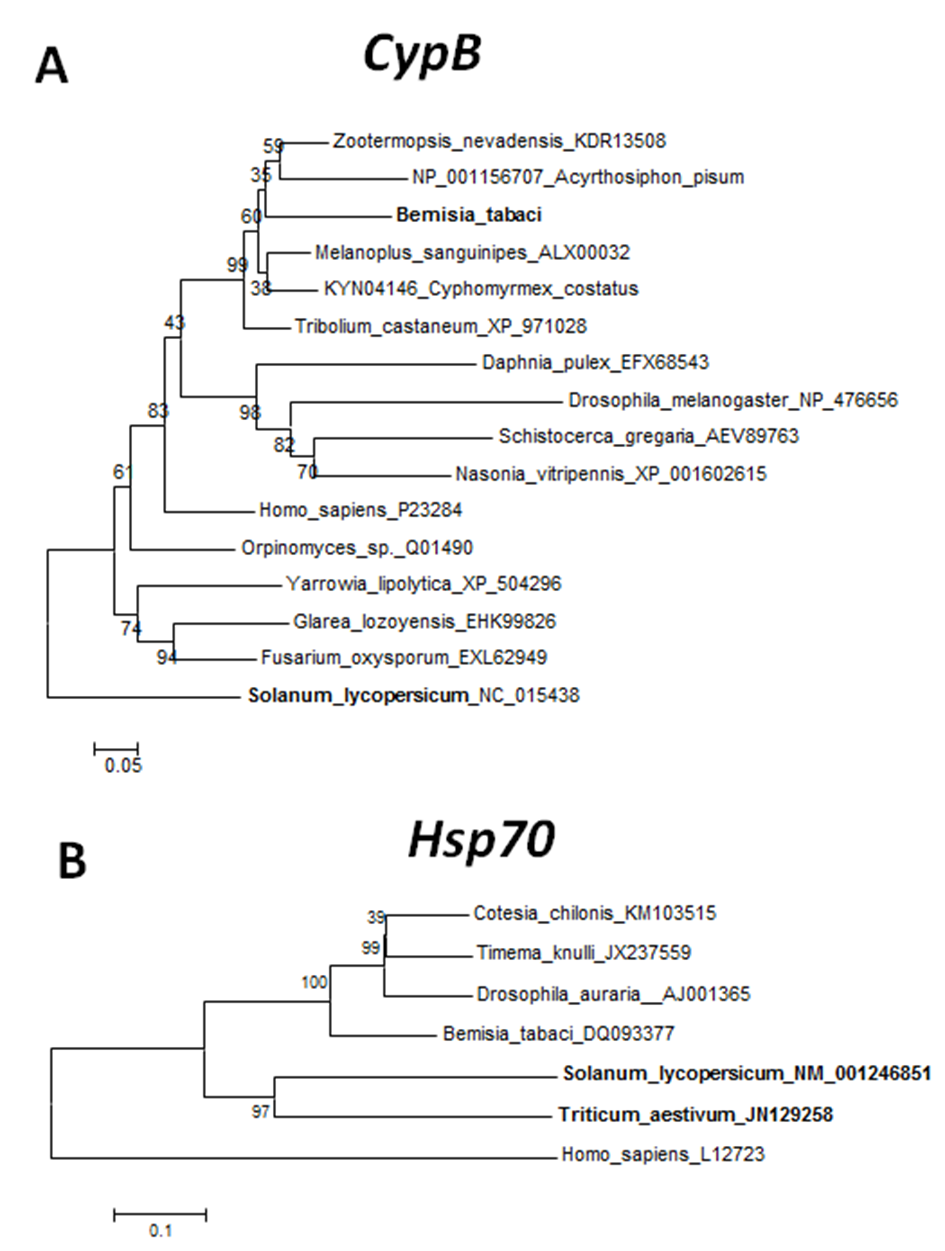

Supplement: FIGURE S2 — Phylogenetic tree of B. tabaci CypB (A) and hsp70 (B) and other arthropods, fungal species, Solanum lycopersicum and H. sapiens. Phylogenetic tree was generated using MEGA 6 (Tamura et al., 2013) with maximum likelihood method. Numbers next to the branches indicated bootstrap value of each internal branch in the phylogenetic tree nodes from 1,000 replicates. [file Image_2.TIF]
