# Supplementary material for: Plant-Mediated Silencing of the Whitefly Bemisia tabaci Cyclophilin B and Heat Shock Protein 70 Impairs Insect Development and Virus Transmission
Source: Front Physiol. 2019 May 8;10:557. doi: 10.3389/fphys.2019.00557 (PMC6517521; doi:10.3389/fphys.2019.00557)
Supplement: TABLE S1 — Primers used in this study for PCR and qRT-PCR analysis. [file Data_Sheet_1.PDF]

Supplementary table 1: Primers used in this study for PCR and qRT-PCR analysis.

Gene/Probe	Name	Primer sequence (5'→3')	Expected product size (bp)	Reference
BtCypB	BtcypBF	TG **GAATTC** ACCCGAAAGTTCAGGTCGGA	282	This study
	BtcypBR	AC **GGATCC** TGAAGTCCTTAATCACCCTATGGA		
CypBs	CypBsF	ATGAAGAACCCGAAAGTTCA	216	Kanakala and Ghanim, 2016
	CypBsR	GAAATTCTCGACAGTCTTCG		
BtHSP70	BtHSP70F	AC **GAATTC** TGAAGGAGAACGCACGATGA	315	This study
	BtHSP70R	TT **GGATCC** AAGCAGTAGGACTCGAGGGA		
TomCyp	TomcypBF	CG **GAATTC** GCACTACAAGGGCTCAACCT	302	This study
	TomcypBR	TA **GGATCC** ATCCAACAGCCTCTGCCTTC		
TomHSP90	TomHSP90F	TGCGTTCTTGTATGGAAGTCTGC	77	Moshe et al., 2016
	TomHSP90R	TGGACCACTTAGTCACGACCAATC		
TRV1	TRV1 F	CATGCTAACAAATTGCGAAAGC	305	This study
	TRV1 R	GGGCGTAATAACGCTTACG		
TRV2	TRV2 F	CGCTGTTTGAGGGAAAAGT	400	This study
	TRV2 R	AAACGCCGATCTCAAACAG		
BT-TYLCV-054-1-C2-T3_C02 (EE601811)	BtC02F	ACTTTTTCAACTCGCAGCTCT	81	Mahadav et al., 2009
	BtC02R	AAATTGGCACAACACTGAGCA		
BT-TOMOV-031-1-E6-T3_E06 (EE598476)	BtE06F	GGAAACTCTGGTGACAGTCGG	81	Mahadav et al., 2009
	BtE06R	TCGAGTATGGCGAGAGGGTG		
B. tabaci β -Actin	Fβ-Actin	TCTTCCAGCCATCCTTCTTG	81	Sinisterra et al., 2005
	Rβ-Actin	CGGTGATTTCCTTCTGCATT		
B. tabaci B-biotype	Bem 23F	CGGAGCTTGCGCCTTAGTC	200	De Barro et al., 2003
	Bem23R	CGGCTTTATCATAGCTCTCGT		
TYLCV CP	V61	ATACTTGGACACCTAATGGC	412	Ghanim et al., 1998
	C473	AGTCACGGGCCCTTACA		
